# Assessment of the Tip Position of Central Venous Catheters Inserted Using Peres’ Height Formula

**DOI:** 10.7759/cureus.31988

**Published:** 2022-11-28

**Authors:** Halide H Şahinkaya, Mine Parlak, Zeki T Tekgul

**Affiliations:** 1 Anesthesiology and Reanimation, University of Health Sciences İzmir Bozyaka Education and Research Hospital, İzmir, TUR

**Keywords:** chest x-ray (cx-ray), peres height formula, complication, central venous catheter (cvc), carina

## Abstract

Objectives: The tip of a central venous catheter (CVC) should be positioned in the proximity of the cavo-atrial junction (CAJ) where the lower third of the superior vena cava (SVC) and the upper right atrium (RA) are located to prevent life-threatening complications. This study aimed to determine the accuracy of Peres’ height formula in predicting the correct insertion depth of CVC.

Methods: A total of 332 patients were enrolled in this prospective observational study. All CVCs were inserted using Peres’ formula. The ‘correct’ tip position of CVC was the placement of the CVC tip 1 cm above and 1 cm below the carina in CXR. Rates of correct placements for each side and site of catheter insertions, gender, and body mass index (BMI) differences were evaluated.

Results: The correct placement rate of all catheters was 74.4%. There were statistically significant correlations between the correct placement of right-sided jugular and subclavian catheters (p<0.001) and left-sided jugular and subclavian catheters (p=0.014). There was a statistically significant difference in male patients (p=0.047). Higher BMI resulted in a lower rate of correct placement with no statistically significant difference (p=0.457).

Conclusions: Peres’ formula can be easily used to determine the correct position of CVC tips with a success rate in the Turkish population. However, practitioners should be aware of the low accuracy rate of Peres’ formula in female patients (68.5%) and patients with BMI over 35 kg/m^2 ^(62.5%).

## Introduction

Central venous catheters (CVCs) are inserted in many critically ill patients for parenteral nutrition and antibiotic therapy as well as patients who require chemotherapy, hemodialysis, patients with difficult peripheral venous access, and surgically ill patients for blood transfusion [1].

It is important to place the catheter tip in the correct position to prevent life-threatening complications such as arrhythmias, and erosion of the right atrium (RA) or right ventricle leading to hemothorax, hydrothorax, or cardiac tamponade. Perforation of the superior vena cava (SVC) can be seen especially during the insertion of left-sided CVCs [2]. The optimal position of the tip also provides the correct measurement of central venous pressure. The tip of a CVC should be positioned in the proximity of the cavo-atrial junction (CAJ) where the lower third of the SVC and the upper RA is located [3]. This position is above the level of pericardial reflection [4]. This is a safe area where the complication rate decreases. A ‘high’ tip position site is on the upper or middle third of the SVC and is related to pain during injection of drugs, thrombosis, and infection. A ‘low’ tip position site is on the inferior vena cava or through the tricuspid valve or the right ventricle. It is also recommended that the catheter tip should not touch the vein wall [5].

There are no gold standards for estimating the insertion depth of a CVC. It is essential to check the correct position of the catheter tip before use. The assessment of the tip of CVC can be performed during and after the insertion. Surface landmarks, simple formulas, fluoroscopy, right atrial electrocardiography (ECG) and ultrasonography (USG), as well as transthoracic echocardiography (TTE) and transesophageal echocardiography (TEE), can be used during the procedure [6-10].

The present study aimed to determine the accuracy of Peres’ height formula in predicting the correct insertion depth of CVC.

## Materials and methods

After obtaining approval from İzmir, Bozyaka Education and Research Hospital Ethical Committee (approval number: 04.07.2018-6), 332 patients within a one-year period, aged between 18 and 70, were enrolled in this prospective observational study. The patients provided written informed consent. Patients with a history of previous neck surgery, abnormal anatomy of the neck, local infection of the neck, chest deformities (e.g., pectus carinatum, pectus excavatum), pacemakers, and coagulopathy were excluded.

Age, gender, weight (kg), height (cm), and body mass index (BMI) (kg/m^2^) were recorded as demographic data. Patients were divided into four groups according to the BMI: BMI Group 1: 18.5- 24.9 kg/m^2^, BMI Group 2: 25- 29.9 kg/m^2^, BMI Group 3: 30.0- 34.9 kg/m^2, ^and BMI Group 4: ≥35 kg/m^2^. All CVCs were placed with aseptic precautions, using the Seldinger method by an intensivist in the intensive care unit (ICU) or an anesthesiologist in the operating room (OR). Peres’ formula was used for the depth of insertion. According to this formula, the lengths for CVC insertion should be height/10 cm for the right internal jugular vein (IJV), (height/10) - 2 cm for the right subclavian vein (SCV), (height/10) + 4 cm for left IJV and (height/10) + 2 cm for left SCV. Triple-lumen catheters (Arrowg+ard Blue® Three-Lumen CVC, Teleflex Medical, Westmeath, Ireland) were used. The patient’s head was turned to the opposite side from the site of insertion. The insertion point was at the level of the cricoid cartilage, and the level of the apex of the two heads of the sternocleidomastoid muscle when IJV catheterization was performed. The SCV catheterization was performed through an infraclavicular approach at the midpoint of the clavicle. Side (right/left) and site (IJV/SCV) of insertion, number of insertion attempts, and complications such as arterial puncture, hematoma, arrhythmias, pneumothorax, and subcutaneous emphysema were recorded.

Postprocedural anteroposterior (AP) CXR was taken in a supine position with the patient’s arms bedside of the chest to assess the CVC tip position and complications. The CXR images were assessed from the Picture Archiving & Communication System (PACS) with the measurement of the vertical distance between the CVC tip and the carina by a radiologist.

The definition for the ‘correct’ tip position of CVC was the placement of the CVC tip 1 cm above and 1 cm below the carina. If needed, the catheter was repositioned by pulling back or changing the catheter through a guide wire for a further distance. The CXR was also used for the correct placement of the CVC tip after repositioning. The primary endpoint was defined as the need for catheter repositioning.

Statistical analyses were performed with the Statistical Product and Service Solutions (SPSS), version 21.0 (IBM Corp., Armonk, NY, USA). Demographic data had a non-normal distribution, so presented as the median (interquartile range (IQR)). Catheter insertion sides, complications, correct placement of CVC tips, and repositioning of catheters were presented as numbers (n) and percentile (%). Correlations between the correct tip position and insertion side, gender, and BMI were determined using the chi-squared test. A p-value lower than 0.05 was considered statistically significant.

## Results

A total of 332 patients were enrolled in the study. There were 202 (60.8 %) male and 130 (39.2%) female patients. Demographic data are listed in Table 1.

**Table 1:** Demographic data Min: Minimum, Max: Maximum, BMI: Body mass index, IQR: Interquartile range

	Min to Max	Median	IQR
Age	18-70	65.5	20
Height (cm)	150-198	168	14
Weight (kg)	45-150	73	13.75
BMI	18.3-66.7	25.59	4

The number of CVCs inserted through the right IJV was 157 (47.3%), and 163 (49.1%) catheters were inserted through the right SCV. The number of catheters placed in the left IJV and left SCV was the same i.e., six (1.8%). A total of 247 (74.4%) CVC tips were in the correct position. The number and percentile of correct placement of CVCs according to the side and site are shown in Table 2.

**Table 2:** Summary of catheterizations and correct tip positions IJV: Internal jugular vein, SCV: Subclavian vein

	Number of catheterizations (n)	Percentage of catheterization (%)	Number of correct tip positions (n)	Percentage of correct tip position (%)
Right IJV	157	47.3	133	84.7
Right SCV	163	49.1	106	65
Left IJV	6	1.8	6	100
Left SCV	6	1.8	2	33.3
Total	332	100	247	74.4

**Figure 1:**
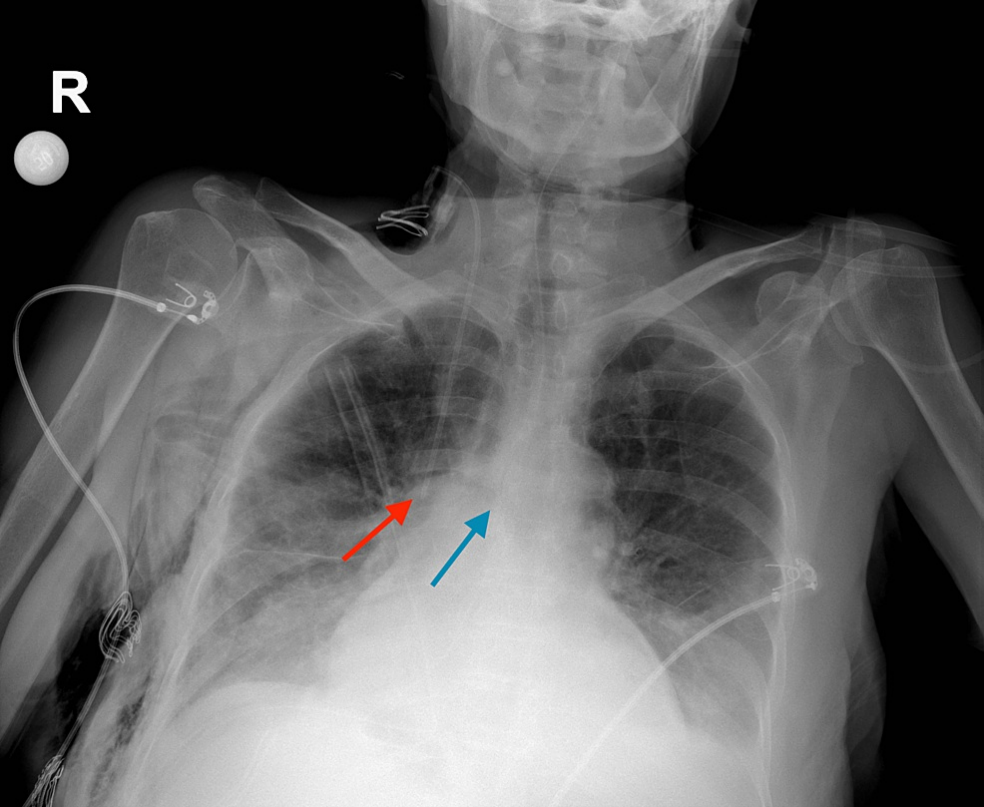
Correct placement of right internal jugular vein catheter Red arrow: Tip of the catheter, Blue arrow: The carina

**Figure 2:**
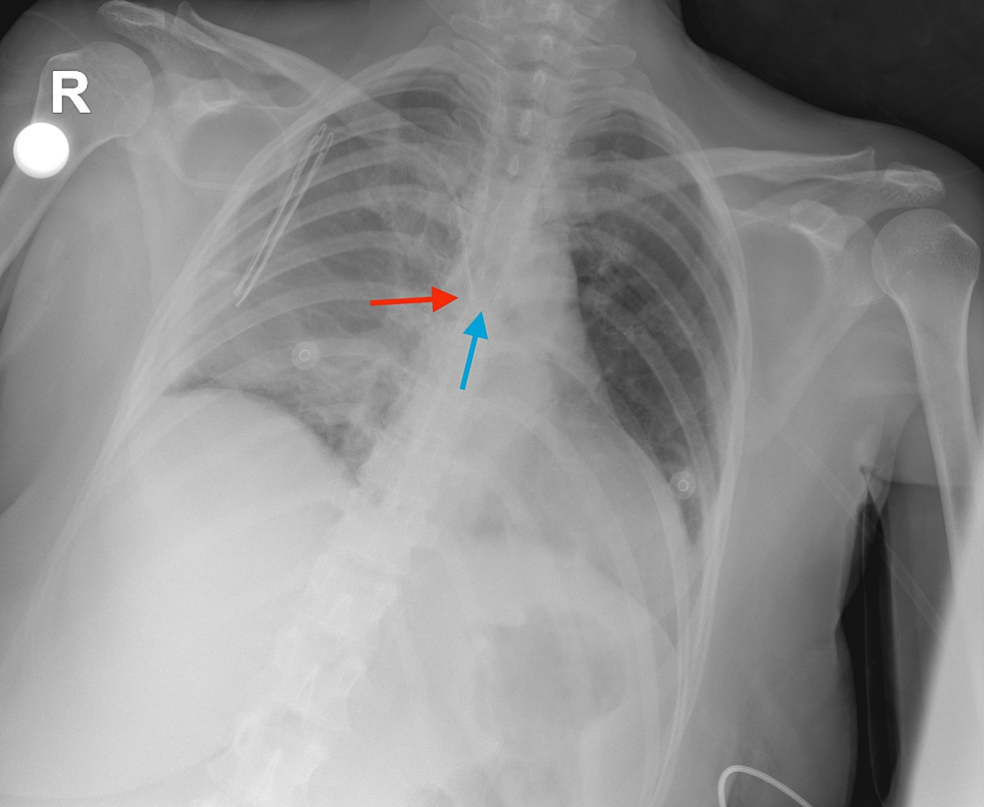
Correct placement of right subclavian vein catheter Red arrow: The tip of the catheter, Blue arrow: The carina

Correct placements for the right IJV and SCV are shown in Figure 1 and Figure 2.

**Figure 3:**
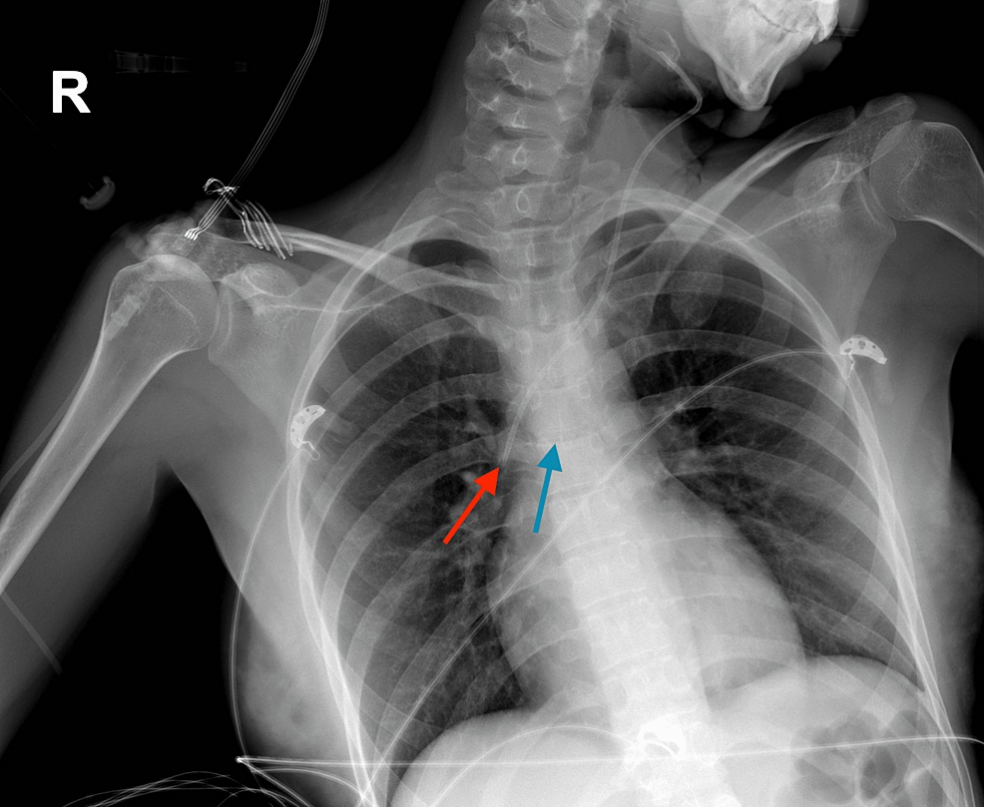
Incorrect placement of a left internal jugular vein catheter Red arrow: The tip of the catheter, Blue arrow: The carina

There were statistically significant differences between the correct placement of right IJV and SCV catheters (p < 0.001) and left IJV and SCV catheters (p=0.014). The number of left IJV and SCV catheters was low. So, the difference between the correct placement of the left IJV and left SCV catheters was thought to be insignificant although the p < 0.001. A total number of 85 (25.6%) catheters were repositioned, where 70 (21.1%) catheters were pulled back and 15 (4.5%) catheters were reinserted for further distance. The incorrect placement of a left IJV catheter, which was located more than 1 cm below the carina, is shown in Figure 3. Postprocedural CXR images of all repositioned CVCs demonstrated correct placement.

The rate of incorrect placement of CVC tips was 31.5% (n=41) in female and 21.8% (n=44) in male patients. There is a statistically significant difference between the correct placement of CVC in male patients compared in female patients (p=0.047). It was seen that 33 (75%) of 44 catheters in male patients and 37 (90.2%) of 41 catheters in female patients were pulled back. The rates of reinsertion to a further distance of CVCs in male and female patients were 25% and 9.8% of all repositioned catheters, respectively.

There were 16 patients with BMI over 35 kg/m^2^. The CVC tips of six (37.5%) of these 16 patients were in the incorrect position. There is not a statistically significant correlation between the correct placement of CVCs and BMI (p=0.457).

Arterial puncture occurred during the placement of 4 (1.2%) catheters, whereas arrhythmias were detected during insertion in 35 (10.5%) patients. Pneumothorax, subcutaneous emphysema, and hematoma were not seen.

## Discussion

The results of the study, where Peres’ formula was used for the insertion depth, showed that 74.4% of all CVC tips were placed in the correct position in the Turkish population. The success rates are 84.7% for the right IJV and 65% for the right SCV. These results are lower than the results in Peres’ study. This difference was thought to be due to the anatomical variations among populations.

Peres first described the formula based on the patient’s height in 1990 [7]. Recommendations for the length of catheter insertion vary with the site and side of insertion. It is recommended that the right SCV catheters should be inserted to a height of 10 cm to 2 cm. The recommended length of the right IJV catheter is a height of 10 cm. Peres has noted that no recommendation can be made for left IJV and left SCV catheters because of the small numbers. The formula also does not take into account the insertion point, anterior, posterior, or central approach for IJV catheterization and as well for supra/infraclavicular SCV catheterization.

Kim et al. recommended a calculation about depths of CVC insertion: 14 cm for the right SCV, 15 cm for the right IJV, 17 cm for the left SCV, and 18 cm for the left IJV, based on the distance from the CVC insertion point to the RA/SVC junction for Asian populations [11]. They used computed tomography (CT) and compared their recommended calculation with Peres’ formula. Peres’ formula predicted the optimal position of CVCs with an accuracy of 75% for the right IJV and 62% for the left IJV. They found that the calculations are more accurate (89% to 95%), more simple to use, and need less repositioning. In the present study, a similar accuracy was demonstrated: 84.7% for the right IJV and 65% for the right SCV. This accuracy is thought to be due to the similarity of the Asian population’s mean height with that of Turkish subjects. Kujur et al. suggested an insertion depth of 16 cm may be adequate for the western population regardless of site and side of insertion, but the Indian population may require a shorter length of placement [12]. Russel et al. suggested a shorter length of 13 cm for the CVCs to be appropriate [13].

Complications such as arrhythmia, sepsis, vessel perforations, cardiac tamponade, embolism, thrombosis, hydropneumomediastinum, and pneumothorax have been reported due to incorrect placement of CVCs [14,15]. In the present study, none of the patients demonstrated pneumothorax or other life-threatening complications. The CVCs were not evaluated for thrombotic and infectious complications in long-term follow-up. Peres also noted low complication rates, with only malposition and arterial puncture [7].

There have been many studies for optimal positioning of CVC tips to prevent complications. The most effective method has been reported to place the CVC tip in SVC, out of the heart, and to check the correct placement with chest radiography [4]. The SVC-RA junction is considered the optimal zone. When the catheter tip is in the upper or middle SVC, thrombosis and migration of the catheter tip into an azygous or innominate vein can be seen. When the catheter tip is in the RA, arrhythmias and cardiac tamponade are the main problems [3].

Manudeep et al. compared the insertion depth of right IJV catheters between the Peres’ formula and the formula calculated from the chest radiography [16]. The distance from the entry point to the midpoint of the right sternoclavicular junction was added to the distance from the midpoint of the right sternoclavicular junction to the carina. This radiological landmark formula was found better than Peres’ formula for optimal depth of insertion. It also takes into consideration the insertion point and anatomical landmarks. Vinay et al. reported that the surface landmark method is superior to the formula approach for the optimal depth of insertion [17].

Kang et al. investigated the tip-to-carina (TC) distance on chest CT and CXR images as many studies have studied [18]. They have demonstrated CT distance as a simple and precise method to confirm not only the correct placement of the CVC tip but also its optimal position for accurate hemodynamic monitoring.

In a recently published study, a modified surface measurement method was used to determine the catheter tip position of a totally implantable venous access port through the right subclavian vein [19]. The distance from the puncture point to the middle point of the sternal notch was added to the distance from the middle point of the sternal notch to the middle point of the Louis angle. The optimal position rate on CXR was found to be 97% while the optimal catheter length rate was 92.4%.

Opposite to all other studies, Struck et al. reported that all calculated formulas had a low likelihood of atrial positions but high risks of proximal mal-positioning [20]. Thus, they did not recommend formulas, considering inter-individual differences in vascular anatomy.

Intra-atrial ECG and detecting the P-wave on the ECG monitor are useful for positioning CVCs. Gebhard et al. reported significantly higher success rates for ECG-guided CVC placements [8]. This method is reliable, prevents radiologic exposure, and minimizes cost. However, it can not be easily used when the P-wave is not detected. It also needs an additional accessory which is not easily available.

Albrecht et al. reported that the upper limit of the pericardial sac is 0.8 cm below the carina in fresh and preserved cadavers [21]. Another study by Dulce et al. studied cadavers and also reported a similar finding [22]. The pericardium can not be seen on a CXR, but the carina can be easily identified. Hence, carina has been considered a radiological landmark for CVC tip position [5].

The CXR is usually preferred to determine the correct position of the CVC tip. Radiation exposure is the main concern about CXR imagining. It is generally used post-procedural, so extra time is needed. It can be available during the procedure only with portable X-ray devices or in catheterization laboratories. Topographic methods are thought to be more reliable to determine the correct positioning as there is no need for extra equipment and cost.

The USG is a cost-effective, intraprocedural, and real-time imagining. Chui et al. reported that a routine postprocedural CXR is unnecessary after ultrasound-guided CVC insertion as pneumothorax and catheter misplacement were rare [23]. Ultrasound guidance is associated with an increased first-attempt success rate, a reduced number of puncture attempts, and fewer complications when compared with the landmark technique [24]. Micro-convex probe is one of the probes that have been used. However, it is feasible only during internal jugular cannulation in adult patients [25]. Disadvantages of the USG technique, including TEE, are the requirement of special equipment and a trained practitioner.

The AP CXR was preferred to confirm the correct position of the CVC tips in this study. The CXRs were examined after the insertion of CVCs by a radiologist. All measurements were made using PACS.

There are some limitations in this study. Firstly, CVCs were inserted by different specialists. Although insertion points were determined by the surface landmarks, these points may have varied according to the practitioner and patient’s head position during insertion. Secondly, CXR images were evaluated after the procedure. There have been other studies where CT imagining was used [11]. The tip of the CVC may have migrated during the transport period. The patient’s position was not checked during imagining as well. Over-extension of the head causes a cephalad position of the tip of CVC. Although the results were statistically analyzed for gender and BMI, age was not taken into account. On the other hand, rates of correct insertion of left-sided catheters did not reflect the real rates due to the small number of patients. Further studies should be planned in a large number of patients with a BMI over 35 kg/m^2^.

## Conclusions

Each catheter tip should be confirmed with an imagining method for correct placement. Development in the field of radiology has led to high-quality imagining methods. However, providing the required equipment and trained practitioners are the main difficulties. So, CXR is still useful for the determination of the CVC tip position. Peres’ formula can be easily used for the correct position of CVC tips with a success rate of 74.4% in the Turkish population. Practitioners should be aware of the low accuracy rate of Peres’ formula in female patients (68.5%) and patients with BMI over 35 kg/m^2^ (62.5%). Further studies with other measurements and different patient groups are needed to achieve higher success rates.
